# Exploring Neighborhood Level Socioeconomic Status and Volumetric MRI Results in Patients with Memory Impairment

**DOI:** 10.1002/alz.089348

**Published:** 2025-01-09

**Authors:** Hannah M Stamos, Mia Achitoov, Nadia Zia, Hessam Sadatsafavi, Bahar Niknejad, Hamid Okhravi

**Affiliations:** ^1^ Eastern Virginia Medical School, Norfolk, VA USA; ^2^ Johns Hopkins University, Baltimore, MD USA

## Abstract

**Background:**

This study addresses the impact of neighborhood‐level socioeconomic disadvantage, as measured by the Area Deprivation Index (ADI), on individuals with mild cognitive impairment and dementia. Our primary outcome aimed to explore associations between ADI and hippocampal volume in cognitively impaired individuals from a comprehensive memory center.

**Methods:**

We conducted a retrospective, cross‐sectional analysis of patients over 50 from Virginia and North Carolina, with visits between January 1st, 2014, and July 1st, 2022. Normative percentile of hippocampal volume, total hippocampal occupancy scores (HOC), superior lateral ventricular volume, and inferior lateral ventricular volume of patients living in census block groups at the top and bottom 20th ADI percentiles were compared. We fitted a linear regression model on propensity‐matched data in the two ADI groups, with multiple covariates. We performed g‐computation in the matched sample and included the interaction of ADI category with covariates and the matching weights in the estimation.

**Results:**

A total of 310 patients were included in the analysis, with 8% of the subjects in the high‐ADI category. The actual sample size slightly varied for each outcome after excluding missing records, thus full matching was utilized. The estimated average treatment effect for high versus low ADI groups only reached statistical significance for hippocampal volume with the difference of 13.9% (SE = 5.83, p = 0.01). The difference of 0.7% (SE = 2.11, p = 0.75) for HOC, 0.5% (SE = 4.13, p = 0.90) for superior lateral ventricular volume, and ‐0.2% (SE = 7.18, p = 0.90) for inferior lateral ventricular volume were not statistically significant.

**Conclusions:**

Contrary to expectations, high versus low ADI groups only showed a difference in hippocampal volume, but in the reverse direction. This finding may suggest global effects of the ADI on the brain health, rather than focal effects on hippocampus. Due to the limited size of our sample in the top 20th percentile, further validation of this discovery is necessary through studies employing larger sample sizes.

